# Transforming Growth Factor-Beta Signaling in the Neural Stem Cell Niche: A Therapeutic Target for Huntington's Disease

**DOI:** 10.1155/2011/124256

**Published:** 2011-05-19

**Authors:** Mahesh Kandasamy, Ralf Reilmann, Jürgen Winkler, Ulrich Bogdahn, Ludwig Aigner

**Affiliations:** ^1^Institute of Molecular Regenerative Medicine, Paracelsus Medical University, Strubergasse 21, 5020 Salzburg, Austria; ^2^Department of Neurology, University of Münster Medical School, 48129 Münster, Germany; ^3^Division of Molecular Neurology, University Hospital Erlangen, 91054 Erlangen, Germany; ^4^Department of Neurology, University of Regensburg, D-93053 Regensburg, Germany

## Abstract

The neural stem cell niches possess the regenerative capacity to generate new functional neurons in the adult brain, suggesting the possibility of endogenous neuronal replacement after injury or disease. Huntington disease (HD) is a neurodegenerative disease and characterized by neuronal loss in the basal ganglia, leading to motor, cognitive, and psychological disabilities. Apparently, in order to make use of the neural stem cell niche as a therapeutic concept for repair strategies in HD, it is important to understand the cellular and molecular composition of the neural stem cell niche under such neurodegenerative conditions. This paper mainly discusses the current knowledge on the regulation of the hippocampal neural stem cell niche in the adult brain and by which mechanism it might be compromised in the case of HD.

## 1. Adult Neurogenesis

The renowned Spanish neuroanatomist Cajal stated that “Once development was ended, the founts of growth and regeneration of the axons and dendrites dried up irrevocably. In adult centers, the nerve paths are something fixed and immutable: everything may die, nothing may be regenerated” [1]. Therefore, it has been believed that no new neurons are generated in the adult brain and most of the common central nervous system (CNS) pathologies accompanied by neuronal loss cannot be restored. Amongst them are the well-known ones: Parkinson's disease (PD) accompanied by the degeneration of dopaminergic neurons in the substantia nigra, Alzheimer's disease (AD) with a neuronal loss in the cerebral cortex and certain subcortical regions, and Huntington's disease (HD), which is an inherited disease that degenerates neurons in the basal ganglia. According to the above-mentioned dogma, the vast majority of neurons in the mammalian brain are generated during embryonic development [2, 3]. This statement stands true for most of the regions of the adult brain. However, this doctrine ended in 1965 when newly generated neurons were found in two specific regions of the adult brain: the subgranular zone (SGZ) in the dentate gyrus (DG) generates new granular neurons in granule cell layer (GCL) of the hippocampus and the subventricular zone (SVZ) of the lateral ventricle wall that gives rise to new cells that migrate along the rostral migratory stream (RMS) to become neurons in the olfactory bulb (OB) [4, 5].

## 2. Hippocampal Neurogenesis

The hippocampus is a bilateral structure that plays a major role in processing and storage of new information. In the hippocampus, stem cells are located along the border between the granular cell layer (GCL) and the hilus known as subgranular zone (SGZ), where they produce cluster-forming precursor cells. From there, neuroblasts migrate into the GCL and become fully matured functional neurons, where they extend dendrites into the molecular layer (ML) and launch mossy fibers to the CA3 region [6, 7]. Following the principle “do or die”, the survival depends on how sufficiently the new cells are integrated into the neural circuit [8–10]. From the neural stem cell to the mature neuron, the cells go through defined steps of division, differentiation, migration, and maturation. Using specific markers, it is possible to investigate the stage-specific changes of SGZ neurogenesis in detail [11, 12]. Further, stem and progenitor cells from adult hippocampus produce neurons that generate action potentials, received functional GABAergic and glutamatergic synaptic inputs [13, 14].

## 3. Neurogenesis in the SVZ -RMS-OB System

The newborn neurons generated in the OB originate from the subventricular zone (SVZ) of the lateral ventricle (LV). In the adult brain, newly generated SVZ young neurons migrate along the rostral migratory stream (RMS) and proceed to the OB [15]. These neuronal cells integrate upon their arrival into the OB as specific subtypes of interneurons. These subtypes are GABAergic granule cells, which represent the majority of the new OB neurons and a very small number of dopaminergic periglomerular interneurons [16, 17]. The olfactory granule cells are inhibitory interneurons that make their dendritic connections to the mitral cells and to the middle tufted cells. The periglomerular neurons project their dendrites into the corresponding glomerulus and connect to the incoming olfactory axons from the sensory epithelium. It has been shown that these newly formed neurons are functionally integrated into the synaptic circuitry of the OB [16, 18, 19].

## 4. The Stem Cell Niches in the Adult Brain

The structural and functional maintenance as well as the regenerative potential of most organs depend on a local population of immature cells termed somatic stem cells. In general, stem cells are placed in defined niches or microenvironments, in which they remain quiescent, but where they can be activated to proliferate and to generate a pool of fast dividing, so-called transient amplifying, progenitor cells. They generate lineage-specific precursors, which migrate towards the ultimate destination where they undergo differentiation into an appropriate functionally mature cell type. In the adult brain, new neurons are generated from neural stem and progenitor cells in the hippocampus and in the SVZ-OB system. These neural stem cells (NSCs) have the capacity to proliferate and to self renew giving rise to neurons, astrocytes, and oligodendrocytes. At present, the functional significance of stem cell-derived adult neurogenesis is still under debate, but most studies indicate that adult neurogenesis is involved in learning and memory processes [20–22]. In addition, the presence of NSCs in the adult brain provides the basis for endogenous cell replacement, which could be developed for future therapies in neurodegenerative disease such as HD. Thus, stimulation of endogenous NSC proliferation and functional integration could compensate neuronal loss. Any therapeutic approach that targets the endogenous neural stem cell population requires a fundamental understanding of the molecular regulation of the stem cell niche, in particular in the case of a degenerative microenvironment as it is present in neurodegenerative diseases.

## 5. Regulation of Adult Neurogenesis

Due to the general recognition and acceptance of adult neurogenesis, there has been an immense response from the scientific community, resulting in a large number of studies investigating how neurogenesis is regulated. Adult neurogenesis is a complex multistep process. This process includes proliferation, cell cycle exit, fate determination of adult neural progenitors and their differentiation, maturation, and final integration into the neural circuits [23]. Although the precise mechanisms that generate new neurons in the adult brain remain elusive, a range of environmental, behavioral, genetic, neuroendocrine, neurochemical and growth factors as well as cytokines have been shown to be involved in the regulation of adult neurogenesis. A number of stimuli have been shown to influence neurogenesis: in an enriched environment the animals are kept in housing conditions that are more similar to their natural environment. Such an enriched condition has given rise to increased neurogenesis and seems to play a neuroprotective role for newly generated neurons [24–26]. Similar to enriched environmental conditions, wheel-running physical exercise has also been shown to boost hippocampal neurogenesis drastically through an increasing rate of progenitor proliferation [27, 28]. The animals that were exposed to an enriched environment and physical exercise showed improved motor skills and better performance in learning tasks [24, 27]. Stroke is a pathological situation in which blood supply to the brain is suddenly disrupted. It has been shown that stroke also stimulates the generation of new neurons [29]. Epileptic seizure is another pathological situation, which arises from the abnormal excitation of neuronal networks in the brain. This epileptic pathological process has also been shown to provoke neurogenesis in the adult brain [30].

Besides, it has been shown that neurogenesis in the hippocampus decreases with aging [31, 32]. Stress is a physiological response to any kind of unpleasant events that provoke the hypothalamic pituitary axis (HPA) and raise the release and circulation of adrenal steroid hormones. Adrenal steroids may be one of the most important neurochemical regulators of neurogenesis. An increased plasma level of corticosterone, as it appears as a reaction to applied stress, has negative effects on hippocampal neurogenesis [33–35]. However, this stress-induced inhibition of neurogenesis can be prevented by systemic administration of neuropeptides such as prolactin (PRL) [36].

## 6. Regulation of Adult Neurogenesis by Signaling Molecules

In the mammalian tissue, typical homeostasis requires elaborately balanced interactions between cells and the network of secreted proteins. These reciprocal communications involve various extracellular cytokines acting via specific cell surface receptors. When the balance between the cells and the extracellular communication is dysregulated, pathogenesis can result [44]. Growth factors are capable of controlling cellular proliferation, differentiation, maturation, and survival. Numerous studies have been carried out to demonstrate that stem and progenitor cells in the adult brain respond to growth factors. Intracerebroventricular infusion of epidermal growth factor (EGF) and fibroblast growth factor-2 (FGF-2) increased proliferation in the SVZ of the adult rats brain [12]. Also insulin-like growth factor-1 (IGF-1) seems to be involved in the regulation of adult neurogenesis. Plasma levels of IGF-1 are increased by exercise, and this promotes major increases in GCL precursor proliferation [45]. Moreover, other studies have demonstrated that intracerebral infusion of IGF increases both cell proliferation and neurogenesis in hypophysectomized rats [46]. Like IGF-1, vascular endothelial growth factor (VEGF) also has a stimulatory effect on neurogenesis [47]. Furthermore, a recent report demonstrated that granulocyte colony-stimulating factor (G-CSF) promotes proliferation of neural progenitors [48].

In contrast, members of the family of transforming growth factor beta (TGF-beta) are known to inhibit neurogenesis by blocking the proliferation of precursor cells in the adult brain. Therefore, TGF-betas and their downstream signaling are at the focus of attention to elucidate their involvement in adult neurogenesis. Bone morphogenetic proteins (BMPs) are extracellular signaling molecules that play diverging roles in neuronal development. Generally, the BMP molecules are characterized by their antagonistic action on neurogenesis. Noggin, for example, is a soluble inhibitor for the BMP4 signal that promotes neurogenesis by blocking the BMP4 influence on stem cell proliferation [49].

## 7. Transforming Growth Factors

The TGF gene family expresses a set of structurally and functionally related polypeptides that include TGF-beta1, TGF-beta2, TGF-beta3, the bone morphogenetic proteins (BMPs), and the growth differentiation factors (GDFs) [50, 51]. The TGF-beta name was coined in the year 1981 because of its transforming effect on rat kidney and fibroblast cell lines [52–54]. TGF-betas have been implicated in cell proliferation, differentiation, migration, survival, apoptosis, extracellular matrix (ECM) formation, angiogenesis, metastasis, tumorogenesis, inflammation, and tissue regeneration [51]. TGF-beta1, TGF-beta2, and TGF-beta3 are the highly homologous isoforms of TGF-beta molecules. Each of these three isoform genes encodes an inactive precursor protein. From the 391-amino-acid precursor form of TGF-beta1, the C-terminal 112 amino acids comprise the mature protein. The N-terminal peptide is the prodomain, called the latency associated peptide (LAP). TGF-beta is secreted as a large latent complex composed of the active TGF-beta form covalently bound to LAP, which in turn is bound to a latent TGF-beta-binding protein (LTBP). Since the LTBP is linked to the extracellular matrix (ECM), the entire complex is stored in the extracellular space and provides a source of readily available ligand. Extracellular serine proteases cleave the LTBP and release the active ligand from LAP [50, 55]. The biologically active form of TGF-beta consists of a homodimer built out of two peptides each in size of 12.5 kD, which are linked through disulfide bonds [56, 57].

## 8. The TGF-Beta Signaling Pathway

**Figure 1:**
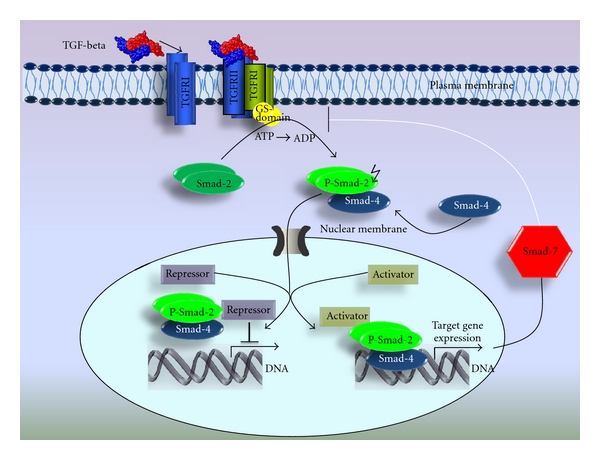
Schematic Illustration: TGF-beta signaling pathway. TGF-beta ligand binds to TGF-betaRII that activates TGF betaRI and induces the downstream Smad-mediated signal transduction.

The TGF-beta family members bind to their cognate heteromeric receptor complex, which consists of two types of transmembrane serine/threonine kinases known as type I (TGF-betaRI or ALK) and type II receptors (TGF-betaRII) [58, 59]. These transmembrane receptors represent two families of serine/threonine kinase receptors of 53 to 65 kD and 80 to 95 kD, respectively. In mammals, five isoforms of TGF-betaRI and seven isoforms of TGF-betaRII were identified. TGF-betaRIII (betaglycan and endoglin) is an indirectly signaling mediator which promotes the affinity of TGF-betaRII for TGF-beta2. In contrast, TGF-beta1 and TGF-beta3 bind directly to TGF-betaRII, a constitutively active kinase that leads to dimerization with the type I receptor and phosphorylation of the glycine-serine (GS) domain. Phosphorylation of the GS domain activates the C-terminal kinase domain, which phosphorylates and thereby activates receptor Smads (homologous proteins to the Sma and Mad proteins from Caenorhabditis elegans and Drosophila melanogaster (R-Smads)). Characteristically, all Smad proteins possess two domains, the MH1 and MH2 (mad homology) domains; the MH1 domain is located on the amino-terminus and the MH2 domain is located on the carboxy-terminus. Functionally, the MH1 is involved in protein-DNA interaction whereas the MH2 is responsible for the protein-protein interaction. Accordingly TGF-beta activates the phosphorylation of Smad2 and Smad3, while BMPs activates the phosphorylation of Smad1, Smad5, and Smad8. The phosphorylated R-Smads dimerize with Co-Smad (Smad4) and transloctate to the cell nucleus where they exert their function as transcription factors. [50, 51, 60]. TGF-beta1 stimulation leads to the nuclear translocation of the phosphorylated Smad 2/3 and of the Co-Smad 4 complex that activates the inhibitory I-Smads (Smad6 and Smad7). These activated I–Smads act as an antagonist for TGF-betaRI-mediated downstream signal by blocking the receptor accessibility to R-Smads [50, 51, 60, 61] (Figure 1). 

## 9. TGF-Beta1 as a Major Regulator of Adult Neurogenesis

Recently, the role of induced level of TGF-beta1 on adult neurogenesis has been described. Thus infusion of TGF-beta1 into the ventricles of the adult rat brain revealed a reduced amount of proliferating cells in the hippocampus and in the SVZ. Further, infusion of TGF-beta1 lowered the number of DCX expressing neuronal precursor in these neurogenic niches. This reduced level of proliferation is strongly correlated with an increased accumulation of phospho-Smad2, an effector of TGF-beta signaling in Sox2/GFAP-expressing cells of SGZ in the TGF-beta1 infused brains [62]. Besides, in an in vitro study, treatment of TGF-beta1 in the neurosphere cultures reduced the proliferation of stem cell and progenitor cells and induced a shift to G0 phase of the cell cycle [62]. Subsequently, a study from the Wyss Coray group has confirmed these findings in the brains of transgenic animals that overexpress TGF-beta1 under the control of the glial fibrillary acidic protein (GFAP) promoter in astrocytes [63]. Besides, other reports mainly focused on the late stages of adult neurogenesis and describe that TGF-beta1 facilitates neuronal differentiation and promotes neuronal survival [64–66].

## 10. TGF-Beta Expression in the Physio- and Pathological Brain

TGF-betas are involved in various physiological and pathological processes in the CNS. All three isoforms of TGF-beta are expressed within the nervous system, in neurons and in glial cells [51, 67, 68]. Most of the current knowledge about the expression of TGF-beta in the CNS comes from studies of the development. In the adult, TGF-beta2 and TGF-beta3 can be found in all areas of the CNS [51]. TGF-beta1 is widely expressed in the choroid plexus and in the meninges, and its expression is drastically upregulated, in the CNS during injury and neurodegeneration [69–72] where it is secreted predominantly by activated microglial cells [73]. In addition, cultivated neurons and astrocytes have been shown to secret TGF-beta1 [74]. In brain pathology, TGF-beta1 is involved in coordinating the inflammatory responses and brain recovery. TGF-beta1 and TGF-beta2 are also involved in brain-tumor development and progression, in particular of high-grade gliomas [51, 75–79].

## 11. Elevated TGF-Beta1 Level and Impaired Neurogenesis in Neurodegenerative Disorders

Neurodegenerative disorders are devastating hereditary and sporadic conditions which are characterized by progressive loss of neuron structure and function, ultimately leading to the death of selective neuronal populations in specific brain areas. Many neurodegenerative disorders occur as a result of degeneration of neurons due to the toxicity of protein aggregation. So far, no promising treatments are available to eradicate these disease conditions. During past decades, series of reports have demonstrated impaired neurogenesis in the brain under degenerative conditions occurring with diseases such as AD, PD, and HD [40, 80, 81]. Therefore, understanding the regulation of neurogenesis in degenerative brains is of crucial importance for therapeutic intervention. In most of the neuropathological conditions, it has been shown that specifically the inflammatory cytokines and their downstream signaling are altered [82]. For example, while neurogenesis is impaired in the diseased brain of patients with AD and HD, the pleiotropic cytokine TGF-beta1 and their downstream signaling components are elevated [72, 83]. This alteration in cytokine expression and its subsequent signaling cascades might be playing a crucial role in impaired neurogenesis.

## 12. Huntington's Disease

Huntington's disease (HD) is an inherited autosomal dominant disorder resulting from an expansion of the CAG repeats within the Huntington gene (HD or HTT) located on chromosome 4 [84]. Healthy individuals have 10 to 35 CAG segment repeats in the HD gene. Individuals with 36 to 40 CAG repeats may or may not develop the signs and symptoms of Huntington's disease, while people with more than 40 repeats have an almost 100% possibility to develop the disorder [85, 86]. The huntingtin protein has very rare homology to other proteins and its functions are poorly understood. The expansion of the CAG repeats causes polyglutamine stretches in the huntingtin protein inducing progressive neurodegeneration [87]. The dysfunction or loss of neurons in the HD brain starts in the striatal region. The striatum is the part of basal ganglia that contains medium spiny neurons (MSN) [88]. In the HD brain, medium spiny neurons are most severely affected resulting in atrophy of the striatum, first in the caudate nucleus, then in the putamen. The second hotspot of neurodegeneration in HD is the cortex. Neurons in layers VI and V of the cortex projecting to the striatum are mostly affected. Furthermore, hippocampal atrophy is typically observed in HD and is correlated with cognitive deficits and depression presented in HD patients [89, 90]. As a consequence of neuronal loss in HD, motor functions and cognition are impaired [91, 92]. Thus far there are no satisfactory therapies available to alleviate this devastating disease. 

The huntingtin protein is widely expressed within the body with the highest levels in the brain and the testis. Within the brain, the highest levels of expression are found in the cerebellar cortex, the striatum and the hippocampus [93, 94]. While the direct function of the huntingtin protein is not yet known, it is apparently required for normal embryogenesis, since HD knockout animals die at an early developmental stage [95]. Conditional knockout studies have demonstrated that the huntingtin protein plays an essential role during postnatal development, as the inactivation of the HD gene in the brain and in the testis leads to degeneration of these two organs [96]. Most importantly, the huntingtin protein is required for neuronal survival [96–98]. This effect is most likely mediated through upregulation of brain-derived neurotrophic factor (BDNF) expression [99]. A recent report indicated that the huntingtin protein is localized at spindle poles during mitosis. Silencing of the HD gene disrupted the spindle orientation and promoted neuronal differentiation of cortical progenitors in mouse embryos [100] highlighting the role of huntingtin in neuronal differentiation.

## 13. Mutant Huntingtin Protein and Intracellular Dysfunctions

In the HD gene, the number of CAG repeats plays a critical role for its pathogenic activities. More than 40 CAG repeats in the HD allele definitively lead to an incorrect folding of the protein, to loss of function and toxic protein aggregation. The mechanism of polyglutamine expansion and its pathogenic roles are unclear. Their direct effect on the neurodegeneration is still under debate, as both defensive and toxic functions have been described. It has been proposed that misfolded huntingtin aggregates translocate to the nucleus, where they form neuronal inclusions (NI) and induce caspase-mediated apoptotic cell death pathways [101, 102]. NIs may interfere with the expression of genes, which are essential for neuronal survival signaling pathways. Recent studies also have shown that mutant huntingtin protein can trap some proteins and dislocate them from their original locations thus interfering or preventing them from their physiological functions [103]. For example, mutant huntingtin protein interferes with the function of cAMP response element-binding (CREB) protein, an important regulatory molecule that is essential for neuronal survival [104, 105]. In addition, mutant huntingtin protein interferes with the ubiquitin proteasome system (UPS), which is in charge of eradicating ubiquitin tagged misfolded or dysfunctional proteins by proteolysis [106–108].

## 14. Experimental Models of Huntington's Disease

### 14.1. Acute Models for Huntington's Disease

Injection of amino acids such as N-methyl- D-aspartate (NMDA), quinolinic acid (QA), or 3-nitro propionic acid (3-NP) leads to neuronal loss in the desired brain region [109–111]. Even though these models display a robust neuronal loss, their value is limited since they do not mirror the genetic component of HD disease. Thus, the following chapter will focus on some of the genetic models for HD.

### 14.2. Transgenic Models of Huntington's Disease

For Huntington disease, several transgenic models have been developed in different organisms ranging from nematodes to primates. The nematode Caenorhabditis elegans is the simplest genetic animal model for polyglutamine (PolyQ) neurotoxicity. Here, the N-terminal 171 amino acid fragment of human huntingtin protein containing an expanded polyglutamine tract is expressed in neurons, where it induces neurodegeneration [112]. Also, PolyQ-expressing fruit flies form NIs and undergo a progressive neurodegeneration [113, 114]. A major breakthrough in the field of HD was achieved with the development of transgenic lines that express the exon1 of htt with 115 CAG (R6/1) or 155 CAG (R6/2) repeats. These animals develop progressive behavioural symptoms and an HD-like neuropathology [115]. They display an early onset of HD pathology, have a shorter life span, and die within the first two to four months of age [115]. A yeast artificial chromosome transgenic mouse model of HD (YAC 128), which expresses the full-length human mutant HD gene with 128 CAG repeats, also shows neurodegeneration in the striatum and in the cortex and an HD-like behavior such as motor and cognitive deficits [116, 117]. Moreover, a transgenic rat model of Huntington's disease (tgHD rat) was developed by von Hörsten and colleagues [118]. This tgHD rat carries a truncated huntingtin cDNA fragment encoding for 51 CAG repeats under the control of the rat huntingtin promoter. The tgHD rats suffer from mitochondrial dysfunction and degeneration of MSNs and show a late onset of motor deficits, emotional disturbance, and cognitive decline [72, 118–120]. The tgHD rat model permits a detailed analysis of progressive structural and functional alterations over time. Moreover, it provides a window of opportunity to examine the impact of any therapeutic attempt.

### 14.3. Modulation of the Neural Stem Cell Niche and Neurogenesis in Huntington's Disease

It has been demonstrated that neurogenesis is impaired in many of the neurodegenerative diseases. Impaired neurogenesis has been suggested to play a major role in the disease progression. In Huntington's disease, it has been shown that neurogenesis is reduced in the hippocampus of R6/1 mouse lines [39] but increased in the SVZ of chemical-induced acute models [38] and HD patients [37]. (Table 1). The underlying molecular and cellular events that lead to these differential alterations in neurogenesis in the HD brains are not known. Strikingly, the expression of TGF-beta1 and TGF-beta signaling components is elevated in the degenerating HD brain. Therefore, it can be hypothesized that TGF-beta1 might be involved in the stem cell niche remodelling in HD brains.

**Figure 2:**
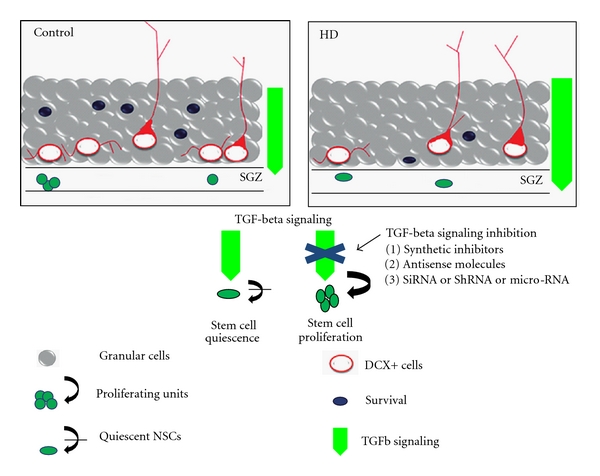
Schematic Illustration: The regulation of neural stem cell niche by TGF-beta1 signaling in tgHD rats. In tgHD rats (HD), the reduced proliferation of NSC (round-shaped green cells) is accompanied by a reduced number of DCX positive cells (red cells) and surviving newly generated cells (round-shaped dark blue cells) compared to control. The number of label-retaining quiescent NSCs (oval-shaped green cells), however, is increased in HD. In control, TGF-beta1 signaling (fluorescence green arrow) is confined to cells of GCL layer (gray cells) and is absent or low in the stem cell niche (SGZ: subgranular zone). In tgHD (HD), however, the overall TGF-beta1 signaling is elevated and in addition, prominent in the stem cell niche, where it accelerates cell cycle exit and NSC quiescence. Thus, inactivation of TGF-beta1 signaling in the stem cell niche might promote NSC proliferation and contribute to neurogenesis.

To investigate the mechanisms that are involved in impairment or modulation of neural stem cell niche in HD, the entire processes of neurogenesis has been explored in the different pathological grade of HD using tgHD rat and R6/2 mouse model. These tgHD models develop progressive cognitive deficits during the disease progression suggesting a possible involvement of hippocampal dysfunction [115, 119]. Recent reports have demonstrated that impaired progenitor proliferation is associated with an increase in neural stem cell quiescence in these transgenic animal models for HD [42, 121]. These observations have also been confirmed in the hippocampus of the YAC128 HD model [122] (Table 1). The tgHD animals encountered a disease-associated progressive decline in hippocampal progenitor proliferation accompanied by an expansion of the pool of BrdU-label-retaining Sox-2 positive quiescent stem cells [121]. Recently, it has been revealed that an elevated level of TGF-beta1 impairs neural progenitor proliferation and induces neural progenitors to exit the cell cycle [62]. Although phospho-Smad2, an effector of TGF-beta signaling, is normally deficient in the stem cell niche, it gradually accumulates in Sox2/GFAP-expressing cells of the subgranular zone in the tgHD brains [121]. Moreover, a comparative transcriptome analysis showed that mRNA expression of TGF-beta1 and its downstream effector molecules were elevated in the human HD brains [72]. This line of evidence points towards the elevation of TGF-beta signaling in tgHD hippocampus, thereby providing an explanation for the reduced NSC proliferation and induced NSC quiescence (Figure 2).

Interestingly, in the early phase of the pathology (i.e., 8 month old tgHD rats), the deficit in NSC proliferation and induced NSC quiescence were compensated by increased DCX-expressing neuroblast proliferation, which resulted in an expansion of the DCX-expressing cell population. Besides survival of newly generated cells, the total number of dentate gyrus neurons and neuronal density were also reduced in tgHD rats and correlated with weaker pCREB signaling [121]. Therefore, the reduction in proliferation might, at least partially, be due to an elevated TGF-beta signaling in the stem cell niche of tgHD animals. Over all, the effect of TGF-beta1-mediated signaling seems to be crucially involved in triggering quiescence of stem cells, as suggested by the accumulation of pSmad2 in Sox2-positive/GFAP-positive SGZ cells (1) in tgHD rats and R6/2 mice [121], (2) after TGF-beta infusion [62], and (3) by the TGF-beta1-induced cell cycle arrest in neural stem and progenitor cultures [62, 121].

## 15. TGF-Beta1 Signaling as a Potential Mechanism Triggering Stem Cell Quiescence to Preserve the Stem Cell Pool in HD

Thus far, the neurodegenerative process-induced quiescence of NSCs in the hippocampal stem cell niche has not been recognized. A number of studies have already examined cell proliferation in the neural stem cell niche of animal models of neurodegeneration and reported reduced progenitor proliferation rates. Hence, this proliferative decline was documented in the R6/1&2 and YAC 128 HD mouse lines [39, 42, 122], in the transgenic mouse models of PD, AD, and ALS [123–125]. A reduction in the numbers of proliferating cells might result from (1) a reduced number of competent NSCs, (2) a prolonged cell cycle, (3) premature differentiation, or (4) a shift of NSCs from the proliferative status to quiescence stage. The most recent findings strongly support the final hypothesis, and it is now crucial to consider the role of TGF-beta1 in the stem cell niche remodelling and induction of stem cell quiescence during the neurodegenerative processes in HD. 

Under normal environment, it is proposed that NSCs limit their mitotic activity and remain mostly in quiescent stage until a self-renewing cycle is required to maintain a steady state pool and to prevent stem cell pool depletion. Acute CNS lesions, such as stroke, QA, and 6-hydroxydopamine-induced striatal atrophies, apparently provide a stimulus to support NSCs proliferation, which is probably in an attempt to compensate for the neuronal loss [38, 126, 127]. Moreover, a recent observation indicated that neuroblast migration is redirected from SVZ-RMS-OB path towards the degenerating striatum of R6/2 mouse [43] (Table 1). On the other hand, slow progressive neurodegeneration often compromise NSCs in their proliferative activity. However, TGF-beta1-induced NSC quiescence might well serve as a mechanism to maintain or to preserve the stem cell pool in the degenerating HD brains. Although the pathways induced by TGF-beta-leading to NSC quiescence require further molecular investigation, mechanisms are likely to be similar to those previously described in different biological systems, in particular in the hematopoietic system. Observation in the hematopoietic system suggested that Pbx1 and Pbx1-dependent genes as well as FoxO3 could be effectors of the TGF-beta-induced quiescence [128, 129]. FoxO3 is a central stem cell maintenance factor integrating a plethora of signaling cascades including the IL-2R/STAT pathway, the TGF-beta/Smad pathway, the PI3K/Akt/mTOR cascade, and Notch signal and is therefore likely involved in the signalling leading to neural stem cell quiescence [130, 131].

## 16. Reactivation of Quiescent Stem Cells as a Regenerative Therapy in Huntington's Disease

Huntington's disease is a progressive neurodegenerative disease for which no complete cure has been established. There are certain drug-based treatments which mainly target reducing the severity of certain symptoms associated with this disease. Tetrabenazine (Xenazine) is the first medication to be prescribed to treat the signs or symptoms of Huntington's disease [132, 133]. This medication helps to reduce the involuntary movements of Huntington's disease by metabolizing the amount of dopamine available in the brain [133, 134]. Also comorbidity of this disease with depression can be treated with drugs such as fluoxetine, sertraline, and nortriptyline [135–137]. However, the side effects of many of these drugs used to treat the symptoms of Huntington's disease may result in further complications rather than cure.

Recent developments in gene silencing technologies such as RNAi and antisense therapy are considered to play a major role in reducing the expression of the misfolded huntingtin protein [138–140]. However, these treatments also have a disadvantage of suppressing the expression of physiological allele of the HD gene. Cells containing the mutant huntingtin protein are known to undergo histone deacetylases-mediated transcriptional dysregulation [104, 141]. HDAC inhibition by HDAC inhibitors might partially restore the transcriptional loss in the HD brain [141, 142]. None of these drugs, however, offer a promising treatment as they involve many side effects and their role in effectively treating this disease still remains debated.

Alternatively, tissue transplantation strategies such as striatal grafts have been proposed as an approach for striatal repair in HD [143, 144]. As a result, this striatal graft onto the brains of transgenic HD R6-lines did not compromise the complete functional outcomes[145]. Further the tissue and cell transplantation strategies exhibit graft rejection problems. Moreover, these transplants may need unique tropic support which might be not supported by the microenvironment in the diseased brain.

Taking the above treatment strategies into consideration, the possibility of endogenous neural stem cells in the stem cell niche of the adult brain would have the potential to compensate and recover neural functions that were lost due to the degenerative processes seen in HD. In the adult brain hippocampal DG provides the niche for stem and progenitors cells and eventually produces new neuron that proposed to compromise the cognitive outcomes. In the HD pathology, neurodegeneration takes place in the hippocampal region but it is inadequately characterized. On other hand, impairment of hippocampal neurogenesis has been clearly demonstrated in most of the models for HD [39, 42, 121, 122]. Thus suggested that there might be a demand of neurons in the hippocampus and that might have an influence on neurogenesis. The stimulation of endogenous stem cell pool would represent a strategy to promote regeneration in the HD brains. However, it has been clearly demonstrated that in tgHD animal brains, upregulated TGF-beta1 and its downstream molecules preserve neuronal stem cells by inducing their quiescent state[121]. Therefore, inactivation of TGF-beta1 signaling specifically in the quiescent stem cells in the stem cell niche of the HD brains would reactivate their proliferation. Eventually, this strategy could compensate the neuronal and functional loss in Huntington's disease. In Huntington's diseased brain, the striatum is the most vulnerable region that is encountered with neuronal dysfunction and neurodegeneration. Persistence of stem cells and its migration capacity in the SVZ region also have a huge potential for replacement therapy. Normally, stem cells proliferate in the SVZ and migrate along the RMS to the olfactory bulb; there they give rise to functional neurons. In pathological conditions like HD, the precursor cells population in the SVZ can be redirected towards adjacent striatum and has possibility for the compensation of neuronal loss [41, 43, 146].

## 17. Conclusion

Recent findings strongly support a hypothesis that defects in progenitor proliferation and an induced NSC quiescence are coordinated by TGF-beta1 signaling in the stem cell niche of Huntington's disease. Thus, TGF-beta1 signaling appears to be a crucial modulator of neurogenesis in HD pathology and it can be a promising target for endogenous cell-based regenerative therapy.

## Figures and Tables

**Table 1:** Adult neurogenesis in HD patients and rodent models.

S.no	HD model	Neurogenic region	Neurogenesis	Reference(s)
1	Human	Subependymal layer	Increased	Curtis et al. 2003 [37]
2	QA-lesioned rat	Subventricular zone	Increased	Tattersfield et al. 2004 [38]
3	R6/1 mouse	Hippocampus	Decreased	Lazic et al. 2004 [39]
				Gil et al. 2005 [40]
4	R6/2 mouse	Hippocampus	Decreased	Phillips et al. 2005 [41]
				Kohl et at. 2007 [42]
5	R6/2 mouse	Subventricular zone	No Change	Phillips et al. 2005 [41]
				Kohl et al. 2010 [43]
6	TgHD rat	Hippocampus	Decreased	Kandasamy et al. 2010 [121]
7	R6/2 mouse	Olfactory Bulb	Decreased	Kohl et al. 2010 [43]
8	YAC 128 mouse	Hippocampus	Decreased	Simpson et al. 2010 [122]
